# A Self-Synthesis Approach to Perceptual Learning for Multisensory Fusion in Robotics

**DOI:** 10.3390/s16101751

**Published:** 2016-10-20

**Authors:** Cristian Axenie, Christoph Richter, Jörg Conradt

**Affiliations:** Neuroscientific System Theory Group, Department of Electrical and Computer Engineering, Technical University of Munich, Arcisstrasse 21, Munich 80333, Germany; c.richter@tum.de (C.R.); conradt@tum.de (J.C.)

**Keywords:** self-construction, self-organization, correlation learning, multisensory fusion, cortically inspired network, mobile robotics

## Abstract

Biological and technical systems operate in a rich multimodal environment. Due to the diversity of incoming sensory streams a system perceives and the variety of motor capabilities a system exhibits there is no single representation and no singular unambiguous interpretation of such a complex scene. In this work we propose a novel sensory processing architecture, inspired by the distributed macro-architecture of the mammalian cortex. The underlying computation is performed by a network of computational maps, each representing a different sensory quantity. All the different sensory streams enter the system through multiple parallel channels. The system autonomously associates and combines them into a coherent representation, given incoming observations. These processes are adaptive and involve learning. The proposed framework introduces mechanisms for self-creation and learning of the functional relations between the computational maps, encoding sensorimotor streams, directly from the data. Its intrinsic scalability, parallelisation, and automatic adaptation to unforeseen sensory perturbations make our approach a promising candidate for robust multisensory fusion in robotic systems. We demonstrate this by applying our model to a 3D motion estimation on a quadrotor.

## 1. Introduction

A fundamental task of a nervous system is to increase the chance of survival of an organism by guiding its actions through the environment, seeking advantageous states, and avoiding dangers. In order to select and execute the most favourable action, either for decision-making [1] or sensorimotor control [2], the system needs information about the dependencies between actions and their consequences. Actions need to be chosen and executed before their consequences can be sensed, such that the system must predict the consequences. This process, associated with the prediction of consequences of actions is partially pre-programmed (i.e., genetically) and partially learned through experience.

Sensorimotor processing is inherently influenced by the real-world constraints and structure. Sensory streams contain certain statistical dependencies determined by the structure of the world, which impose constraints on a system’s sensorimotor affordances [3]. This limits the number of possible sensory information patterns and plausible motor actions. Learning mechanisms allow the system to extract the underlying correlations in sensorimotor streams. This increases flexibility and robustness in the face of uncertainty and in an ever-changing environment.

Today’s technical systems, in particular mobile robots, have limited capabilities to generalize their sensorimotor capabilities to new tasks. Typically, the system designer is responsible for defining the system’s structure and describe its sensorimotor dynamics. Although providing good solutions for particular scenarios, traditional system design approaches are bound to parametrization routines characterised by a high use of computational resources and processing time. Moreover, task-dependent parameter configurations and prior assumptions limit flexibility.

Tackling the challenging problem of learning underlying correlations from data, our approach comes as an alternative to already developed methods. Various methods, ranging from neural circuitry implementations to statistical correlation analysis, have been developed to extract correlational structure in sensory data.

Related work in [4] used a combination of simple biologically plausible mechanisms, such as Winner-Take-All (WTA) circuitry, Hebbian learning, and homeostatic activity regulation, to extract relations in artificially generated sensory data. Using a different neurally inspired substrate, the model developed in [5] combined competition and cooperation in a self-organizing network of processing units to extract coordinate transformations in a robotic visual object localization scenario. Going away from biological inspiration the work in [6] used a nonlinear canonical correlation analysis method (i.e., Alpha-Beta Divergence Correlation Analysis (ABCA)), to extract relations between sets of multidimensional random variables based on a divergence metric. Using an artificial neural network (ANN) for implementing canonical correlation analysis the work in [7] proposed a model able to extract the underlying structures between two sets of variables under moderate noise conditions.

In order to frame our work within state-of-the-art using similar data analysis tools we briefly introduce work carried on sensor fusion using entropy and information theory, e.g., condition monitoring, predictive control. Introducing an extension for Dempster-Schafer evidence theory, the study proposed in [8] intorduced a sensor fusion system by employing an entropy metric for improving accuracy and reliability of the consensus among various sensory quantities in wireless sensor networks. The work employed a distributed computation of entropy and an analysis of global system entropy minimization, similar to our work. The core approach differs from our work which focuses on exploiting the underlying regularities to build sensory associations before the fusion process. Analysing a multimodal sensor fusion scenario, the strudy in [9] used kernel entropy and correntropy for fusing audio and video signals for intelligent human-machine-interfaces providing superior performance over state-of-the-art in terms of accuracy and reliability. Focusing on a high-level (feature) understanding and correlation within multimodal data the approach differs from our proposed technique which focuses on the low-level regularities in the multimodal streams. Focusing on entropy based fusion for condition monitoring, the work in [10] introduced an entropy based sensor selection method for machine condition monitoring and prediction by analysing the statistical regularities and uncertainty in multiple sensory streams. This work focuses on sensor selection as a prior step in modelling the system’s perceived dynamics and differs from our proposed technique which focuses on the low-level regularities in the multimodal streams. Finally, analysing the potential of entropy in optimal control the model developed in [11] employed an entropy metric to characterize the uncertainty of the closed loop system by defining a joint probability performance index in a DC motor control system with non-Gaussian noise and delays. In this study, the entropy metric is optimizing a performance index for a controller and doesn’t exploit the underlying statistics of the data for associating various sensory quantities as shown in our work.

Lessons from neuroscience taught us that learning mechanisms allow a system to generalize over multiple tasks. Perceptual learning is the way developing organisms discover invariants of features, states, and layout of the environment in new contexts [12]. Perceptual learning can be realized through the exploration of the underlying correlational structure in the sensorimotor streams. Exploiting the long-term history of the motor commands and their sensory consequences allows the system to learn and refine an internal model, a process coined structural learning by Braun in [13].

The question is how to engineer a model that has a structure capable of representing the relation between inputs or between inputs and outputs? One approach is to exploit regularities in correlated sensorimotor streams in order to associate relevant cues that disambiguate the system’s state. Is it advantageous to design a system capable to learn its own structure by autonomously extracting correlations in its sensorimotor space? In this work we tackle these questions and show that, once sensory associations are learned, generalization can be conceived as an adaptation of the internal model to the learned structure.

In order to frame our work in the already introduced context, we address the problem of robust self-motion perception. We explore how this task can be performed efficiently through learning sensorimotor correlations. Not only are there many types and combinations of motion, but there are various factors governing the perception of self-motion and orientation, an important one being the functional representation between sensory physics and the nervous system [14]. Adhering to this view, our work investigates neural mechanisms for inferring a structure capable of supporting efficient learning and adaptation in extracting functional representations among sensorimotor streams. The learned representations are subsequently employed in multisensory integration yielding more precise motion estimates of the system.

After briefly describing the investigation context in the Introduction section, we introduce the generic neural processing model in the Methods section. We focus on the basic functionality for correlation extraction, self-synthesis, and the specific tools used in our real-world instantiation. Our preliminary results, along with an in-depth analysis, are introduced in the Results sections. The core observations in the Results section are further extended in the Discussion session where we also introduce alternative approaches and evaluate their applicability in real-world scenarios. Providing a thorough analysis and comparison, this section emphasizes the novelty and the main contributions of our work. Finally, the Conclusions section revamps the core principles and motivation behind our work and provides an outlook on possible extensions and improvements.

## 2. Materials and Methods

The core contribution of this work is a novel processing and representation paradigm capable of extracting underlying correlations in incoming sensory streams and integrating their contributions into a coherent percept. In this work we employ a processing principle known to be ubiquitous in the mammalian cortex, namely distributed processing with only local processing and storage. Obeying local dynamics of mutual interaction, the multitude of sensorimotor streams, encoded as processing maps, converge to global consensus. Aiming at obtaining a globally consistent representation, each map tries to minimize the disagreement among connected representations. Globally, this process learns an internal model based on underlying physical properties of cross-sensory interactions via continuous belief update given sensory observations.

In order to validate our framework and its hypotheses, we extend the basic model in [15] towards perceptual learning for multisensory fusion. Using cortical maps as neural substrate for distributed representations of sensorimotor streams, the system is able to learn its connectivity (i.e., structure) from the long-term evolution of sensory observations. Changing representation, from single point estimates to a sparse encoding of sensorimotor streams, the system exploits the intrinsic correlations in the activity patterns of a network of neural processing units. This process mimics a typical development process where self-construction (connectivity learning), self-organization, and correlation extraction ensure a refined and stable representation and processing substrate, as shown in [16]. Following these principles, we propose a model based on Self-Organizing Maps (SOM) [17] and Hebbian Learning (HL) [18] as main ingredients for extracting underlying correlations in sensory data.

### 2.1. Basic Correlation Learning Model

**Figure 1:**
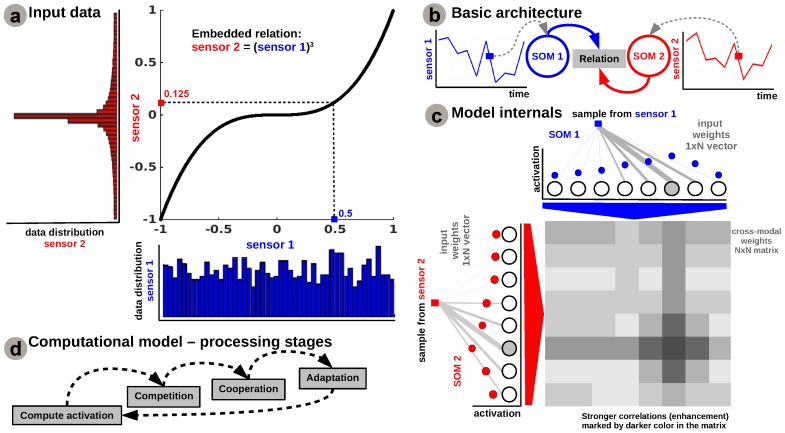
Model architecture. (**a**) Input data resembling a nonlinear relation and its distribution; (**b**) Basic architecture; (**c**) Model internal structure; (**d**) Processing stages.

In order to give an intuition on the inner workings of the aforementioned mechanisms, we start with a simple bimodal scenario, depicted in Figure 1b, in which the correlation among two sensors is represented by a simple nonlinear relation, e.g., power-law, as depicted in Figure 1a.

The input Self-Organizing Maps (SOMs) are responsible for extracting the statistics of the incoming data and encoding sensory samples in a distributed activity pattern, as shown in Figure 1a,c, respectively. This activity pattern is generated such that the neuron closest to the input sample, in terms of its preferred value, will be strongly activated. Activation decays as a function of distance between input and preferred value. Using the SOM distributed representation, the model learns the boundaries of the input data, such that, after relaxation, the SOMs provide a topology preserving representation of the input space. We extend the basic SOM, introduced in [17], in such a way that each neuron not only specialises in representing a certain (preferred) value in the input space, but also learns its own sensitivity (i.e., tuning curve shape). Given an input sample, $s^{p}\left(k\right)$ at time step *k*, the network follows the processing stages depicted in Figure 1d. For each *i*-th neuron in the *p*-th input SOM, with the preferred value $w_{in,i}^{p}$ and $\xi_{i}^{p}\left(k\right)$ tuning curve width, the sensory elicited activation is given by

(1) $$a_{i}^{p}\left(k\right)=\frac{1}{\sqrt{2\pi }\xi_{i}^{p}\left(k\right)}exp\left(\frac{-{\left(s^{p}\left(k\right)-w_{in,i}^{p}\left(k\right)\right)}^{2}}{2{\xi_{i}^{p}\left(k\right)}^{2}}\right).$$

The winner neuron of the *p*-th population, $b^{p}\left(k\right)$, is the one which elicits the highest activation given the sensory input at time step *k*

(2) $$b^{p}\left(k\right)=argmax\;a^{p}\left(k\right).$$

During self-organisation, at the input level, competition for highest activation is followed by cooperation in representing the input space (second and third step in Figure 1d). Similar to the generic SOM model, given the winner neuron, $b^{p}\left(k\right)$, the interaction kernel,

(3) $$h_{b,i}^{p}\left(k\right)=exp\left(\frac{-\left|\right|r_{i}-r_{b}{\left|\right|}^{2}}{2{\sigma (k)}^{2}}\right).$$

allows neighbouring cells (found at position $r_{i}$ in the network) to precisely represent the sensory input sample given their location in the neighbourhood σ(k). The interaction kernel in Equation (3), ensures that specific neurons in the network specialise on different areas in the sensory space, such that the input weights (i.e., preferred values) of the neurons are pulled closer to the input sample with a decaying learning rate α(k),

(4) $$\Delta w_{in,i}^{p}\left(k\right)=\alpha \left(k\right)h_{b,i}^{p}\left(k\right)\left(s^{p}\left(k\right)-w_{in,i}^{p}\left(k\right)\right).$$

This corresponds to the adaptation stage in Figure 1d and ends with updating the tuning curves. Each neuron’s tuning curve is modulated by the spatial location of the neuron, the (Euclidian) distance to the input sample, the interaction kernel size, and the learning rate,

(5) $$\Delta \xi_{i}^{p}\left(k\right)=\alpha \left(k\right)h_{b,i}^{p}\left(k\right)\left({\left(s^{p}\left(k\right)-w_{in,i}^{p}\left(k\right)\right)}^{2}-\xi_{i}^{p}{\left(k\right)}^{2}\right).$$

**Figure 2:**
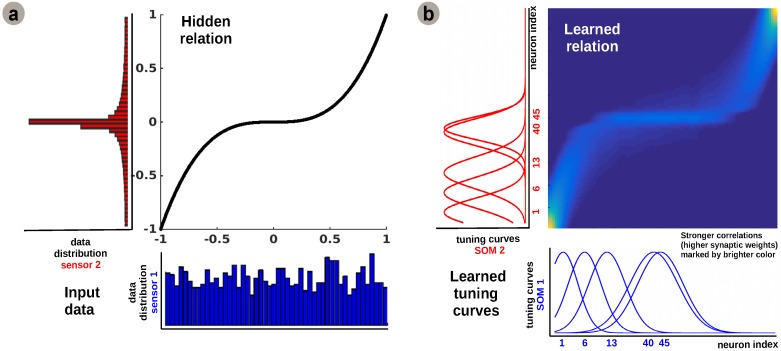
Extracted sensory relation and data statistics using the proposed model: (**a**) Input data statistics and hidden relation; (**b**) Learned preferred values and underlying relation.

The learned tuning curve shapes for 5 representative neurons in the input SOMs (i.e., neurons 1, 6, 13, 40, 45) are depicted in Figure 2b. We observe that higher input probability distributions, as shown in Figure 2a, are represented by a large number of sharp tuning curves, whereas lower or uniform probability distributions are represented by a small number of wide tuning curves.

Using this mechanism, the network optimally allocates resources (i.e., neurons): a higher amount to areas in the input space which need a finer representation; and a lower amount for those areas that don’t. This feature emerging from the model is consistent with recent work on optimal sensory encoding in neural populations [19]. This claims that, in order to maximise the information extracted from the sensory streams, the prior distribution of sensory data must be embedded in the neural representation.

The second component of our model is the Hebbian linkage, more precisely a covariance learning rule akin to the one introduced in [18]: a fully connected matrix of synaptic connections between neurons in each input SOM, such that the projections propagate from pre-synaptic units to post-synaptic units in the network. Using an all-to-all connectivity pattern, each SOM unit activation is projected through the Hebbian matrix. The Hebbian learning process is responsible for extracting the co-activation pattern between the input layers (i.e., SOMs), as shown in Figure 1c, and for eventually encoding the learned relation between the sensors, as shown in Figure 2b. The central panel of Figure 2b demonstrates that connections between uncorrelated (or weakly correlated) neurons in each population are suppressed (i.e., darker color-lower value) while correlated neurons’ connections are enhanced (i.e., brighter color-higher value). The boundary effects are not explicitly handled in the network as they don’t disrupt the overall relation learning process. A simple solution will be to consider a distance metric $\left|\right|r_{i}-r_{j}\left|\right|=min\left(|i-j|,N-|i-j|\right)$, with *i*,*j*-units in the population, that allows wrap-up and a uniform distribution of the activity at the boundaries.

The effective correlation pattern encoded in the $w_{cross}$ matrix, imposes constraints upon possible sensory values. Moreover, after the network converges, the learned sensory dependency will make sure that values are “pulled” towards the correct (i.e., learned) corresponding values, will neglect outliers, and will allow inferring missing sensory quantities. Formally, Hebbian connection weights, $w_{cross,i,j}^{p}$, between neurons i,j in each of the input SOM population are updated using

(6) $$\Delta w_{cross,i,j}^{p}\left(k\right)=\eta \left(k\right)\left(a_{i}^{p}\left(k\right)-{\bar{a}}_{i}^{p}\left(k\right)\right)\left(a_{j}^{q}\left(k\right)-{\bar{a}}_{j}^{q}\left(k\right)\right),$$

where

(7) $${\bar{a}}_{i}^{p}\left(k\right)=\left(1-\beta \left(k\right)\right){\bar{a}}_{i}^{p}\left(k-1\right)+\beta \left(k\right)a_{i}^{p}\left(k\right),$$

and β(k), η(k) are monotonically decaying functions (i.e., inverse time functions) parametrized as:

(8) $$\beta \left(k\right)=0.002+\frac{0.998}{k+2},\eta \left(k\right)=\frac{A}{k+B},B=\frac{v_{f}t_{f}-v_{0}t_{0}}{v_{f}-v_{0}},A=v_{0}t_{0}+Bv_{0},$$

where $v_{0},v_{f}$ are the pre-set initial (time $t_{0}$) and final (time $t_{f}$) values of η(k). Self-organisation and correlation learning processes evolve simultaneously, such that both representation and correlation pattern are continuously refined.

### 2.2. Structure Learning for the Multisensory Fusion Model

**Figure 3:**
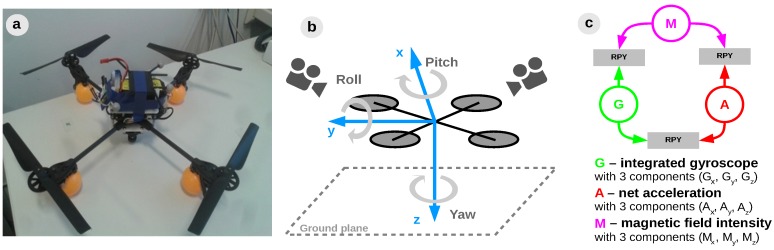
Experimental setup: (**a**) Quadrotor platform; (**b**) Reference system alignment and ground truth camera tracking system; (**c**) Sensors used in the experiement for Roll-Pitch-Yaw (RPY) estimation.

In order to test the proposed model, we apply it for a quadrotor 3D egomotion estimation, as depicted in Figure 3.

This shall serve as an example to introduce the system structure learning mechanism, namely the process that infers a plausible network structure for multisensory fusion. Envisioning a system capable of self-deployment, our work proposes an autonomous method to learn a system’s structure from sensory associations. Prior to learning multisensory fusion rules, the system must decide which sensors can be associated for coherent estimates of each motion component, using only available on-board sensors. The basic idea is to determine which regularities in the different sensory streams are informative and enforce the connections between correlated sensors to provide a relevant rule for fusion. As the system follows a developmental process (offline), one can only evaluate the system’s real-time capabilities after learning. The sensors used in our instantiation are the 3-dimensional integrated gyroscope readings, 3-dimensional net accelerations, and 3-dimensional magnetic field intensities, as shown in Figure 3c. In order to obtain informative quantities into the algorithm we combine the 3 components of each sensor in derived cues relevant for each degree of freedom. For example, we combine acceleration on *x*-axis and gravity (i.e., acceleration on *y*-axis) to extract the pitch contribution of the acceleration, and we combine magnetic field intensities on the x and y axes to extract the yaw contribution of the magnetometer.

Physical systems are continuously and dynamically coupled to their environment. This coupling offers the system the capability to explicitly structure its sensory input and generate statistical regularities in it [20]. Such regularities in the structure of the incoming multisensory streams are crucial to enabling adaptation, learning, and development. Providing a practical approach to measure statistical regularities, dependencies, or relationships between sensory streams, information theoretic measures can be used to quantify statistical structure in real-world data streams, as shown in [21,22].

In our approach we address the problem of recovering the structure of a network from available sensory data in its most general form, namely time-series streams of sensory data. No assumptions about the underlying structure of the sensory data are made and no prior knowledge about the system is taken into account. Furthermore, interactions between the various sensory streams are deduced from the statistical features of the data using information theoretic tools. This approach extends the generality of our framework for learning sensory correlations used for multisensory fusion.

In the simplest bimodal scenario, we assume *X* and *Y* to denote random sensory variables consisting of the set of possible samples $x_{i},y_{i},i=1,...,n$, with associated probability mass functions $p\left(x_{i}\right),p\left(y_{i}\right),i=1,...,n$. An important information theoretic metric, relevant for this problem, is the relative entropy between the joint distribution $p(x_{i},y_{j})$ and the product distribution $p\left(x_{i}\right)p\left(y_{j}\right)$ of the two sensory variables, which defines the mutual information:

(9) $$I\left(X,Y\right)=\sum_{i}\sum_{j}p\left(x_{i},y_{j}\right)log\frac{p(x_{i},y_{j})}{p\left(x_{i}\right)p\left(y_{j}\right)},$$

given that we consider the general form of information entropy,

(10) $$H\left(X\right)=-\sum_{i=1}^{n}p\left(x_{i}\right)log\left(p\left(x_{i}\right)\right).$$

Intuitively, mutual information is high if both sensory quantities have high variance (i.e., high entropy) and are highly correlated (i.e., high covariance) [23]. In our scenario, if two components of the network of sensory variables interact closely (correlated statistical regularities) their mutual information will be large, whereas if they are not related their mutual information will be near zero.

**Figure 4:**
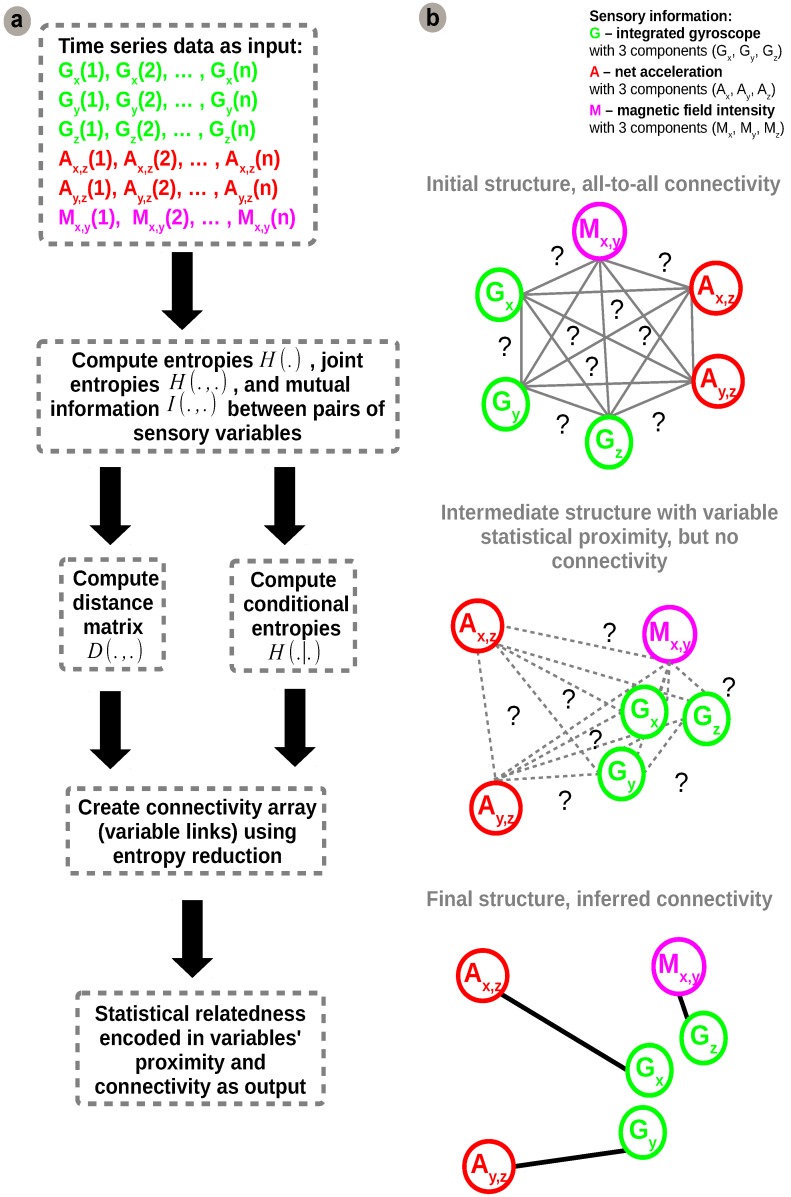
Network inference algorithm: (**a**) Algorithm pipeline: feed time-series sensory input; compute statistics for individual and pairs of sensors (entropy and mutual information); compute statistical distance and conditional entropies to extract statistical relatedness; create connectivity array using entropy reduction (minimisation); (**b**) Network structure evolution: initial connectivity; intermediate statistically clustered variables; final structure and inferred connectivity.

Using this basic formulation of information theoretic metrics, we developed our network inference algorithm which is synthetically depicted in Figure 4.

Initially, uni-dimensional, multi-dimensional (joint and conditional variables) entropies, H(X,Y),H(X|Y), and mutual information measures are estimated from sensory data, as shown in Figure 4a. The estimates are subsequently used for calculating distances between variables and build a distance matrix. In order to discriminate between direct and indirect (implicit) connections an entropy reduction (minimisation) step is applied on conditional entropies, similar to [24].

The distance metric used for constructing the distance matrix is the Entropy Metric Construction (EMC) [25,26]. An important feature of this metric is that it takes into account possible time delays *τ* in the sensory data time-series:

(11) $$d{\left(X,Y\right)}^{EMC}=min_{\tau }\;e^{-I(X(t+\tau ),Y(t))}.$$

**Figure 5:**
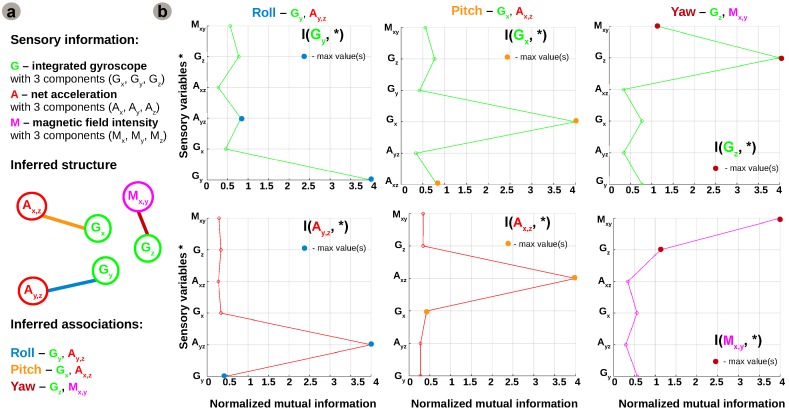
Network inference analysis: (**a**) Sensory data, inferred network structure, and associations for each motion component; (**b**) Individual estimates of mutual information, on a per sensory variable basis, motivating the established network connections for sensory associations.

The sensory readings are coming from different sources but all measure the consequences of the robot motion. Equation (11) provides a generic formulation in which, for example, one sensor can be a delayed copy of another (i.e., measuring the same quantity but with different principle). In our case *τ* is 0, as the sensors are sampled at the same time although, due to physics, they have different reaction time induced by the motion of the robot. It is easy to see that high values of mutual information between variables determine a smaller distance value as shown in Figure 5b. Due to the fact that we need to infer the network structure from sensory data, knowledge about the underlying system cannot be used. Hence, we need to estimate mutual information from the datasets instead of using the analytical form. In order to refine the interactions among sensory variables we use an entropy reduction process that seeks to determine variation in one sensory variable given variation in another sensory variable. The mechanism assumes that if a sensory variable $X^{*}$ is connected to *Y* (which has already been predicted to be connected to a subset $X_{s}^{*}$ of $X^{*}$), its inclusion in the network structure must reduce the entropy by a proportion at least equal to a threshold *T*. The threshold *T* is computed as a function of overall entropy values, a subunit average of all possible combinations of variables in all network configurations (i.e., circular permutations on all available variables). Hence, a link between $X^{*}$ and *Y* is predicted if and only if the entropy reduction $E_{R}\left(Y,X^{*}\right)$, in Equation (12), exceeds the threshold *T*.

(12) $$E_{R}\left(Y,X^{*}\right)=\frac{H\left(Y|X_{s}^{*}\right)-H\left(Y|X_{s}^{*},X^{*}\right)}{H(Y)}.$$

In order to obtain reliable estimates of joint entropies of the many sensory variables, the large amount of data observations ( >13,000 samples) provides an advantage. Furthermore, exploiting the rich input space, the proposed algorithm is able to exploit the intrinsic statistical regularities of the sensory data to generate a plausible network configuration. The raw sensory data fed to the system is sampled at 200 Hz. For each degree of freedom there is decoupled data from each sensor axis paired for each degree of freedom (roll—$G_{y}$ and $A_{y,z}=A_{y}/A_{z}$, pitch—$G_{x}$ and $A_{x,z}=A_{x}/A_{z}$, yaw—$G_{z}$ and $M_{x,y}=M_{x}/M_{y}$). Analysing individual statistics, from the perspective of each variable with respect to all the others, the network configuration generated by the algorithm is supported by the pairs of mutual information estimates depicted in Figure 5.

**Figure 6:**
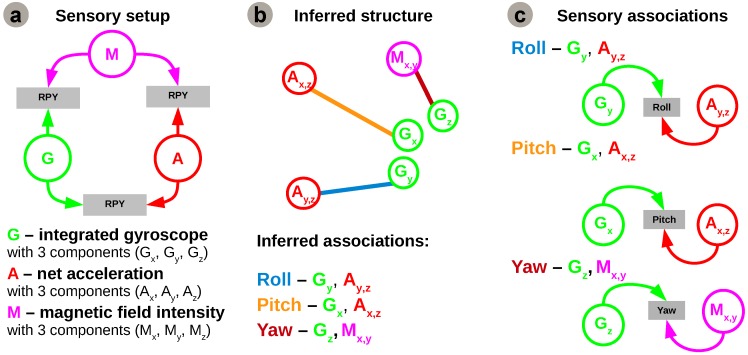
Network instantiation for 3D egomotion estimation: inferred network structure and sensory associations for learning. (**a**) On-board sensory configuration; (**b**) Inferred network connectivity; (**c**) Sensory associations for learning.

Although initially the network considers all sensory contributions for the estimation of all motion components it will enforce only those connections providing a coherent correlation for each degree of freedom based on the resulting configuration from the network inference algorithm. Using only the underlying statistical regularities and information content in incoming sensory streams, the algorithm detects, connects, and combines sensory contributions which are informative for estimating the same degree of freedom into motion estimates, as depicted in Figure 6c.

For roll and pitch angles (i.e., rotation around the x and y reference frame axes), the network learns the relation between the roll and pitch angle estimates from gyroscope data and rotational acceleration components (i.e., orthogonal x and y with respect to z reference frame axes). Similarly, the yaw angle is extracted by learning the relation between the yaw angle estimate from integrated gyroscope data (i.e., absolute angle) and aligned magnetic field components from the magnetic sensor (i.e., projected magnetic field vectors on orthogonal x and y reference frame axes). The learned sensory associations are not arbitrary, but rather represent the dynamics of the system and are consistent with recently developed modelling and attitude control approaches for quadrotors [27,28].

To make use of the learned relations, we decode the Hebbian connectivity matrix using a relatively simple optimisation method [29]. After learning, we apply sensory data from one source and compute the sensory elicited activation in its corresponding (presynaptic) SOM neural population. Furthermore, using the learned cross-modal Hebbian weights and the presynaptic activation, we can compute the postsynaptic activation. Given that the neural populations encoding the sensory data are topologically organised (i.e., adjacent values coding for adjacent places in the input space), we can precisely extract (through optimisation) the sensory value for the second sensor, given the postsynaptic activation pattern. Without using an explicit function to optimise, but rather the correlation in activation patterns in the input SOMs, the network can extract the relation between the sensors.

## 3. Results

By tackling a challenging multisensory fusion scenario for 3D motion estimation, we designed a system capable to extract associations between sensory cues from on-board sensory data without any prior assumptions. The system structure emerges from the underlying regularities in the sensory streams. Once relevant associations are extracted cross-sensory relations are learned. The learned relations constitute the rules to integrate the available on-board sensory quantities. These rules impose constraints on the possible values a sensory cue can take by updating the representations toward a coherent state.

**Figure 7:**
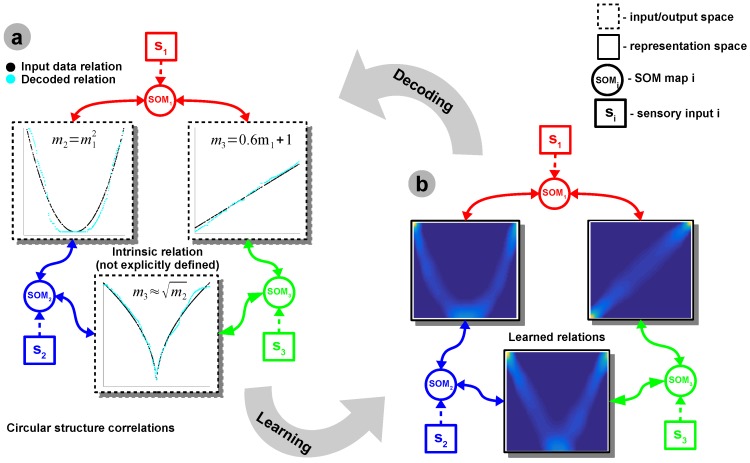
Basic system analysis. Sample scenario with a 3-dimensional network with a circular correlation structure. (**a**) Input data and decoded learned representation: the inputs are encoded in distributed neural activation profiles using Self-Organising Maps (SOM); the temporal coincidence of these activations strengthen the connection weights in the representation space using Hebbian learning (HL); (**b**) Learned relations.

The inferred relations provide a plausible description of cross-sensory interactions and provide prediction capabilities and improved individual sensory estimates for each motion component. This capability is a fundamental strength of the system as it provides a robust way to infer the underlying internal model, while preserving a stable cross-sensory integration mechanism. This behaviour is visible in a sample scenario depicted in Figure 7. Given arbitrary nonlinear relations among the different sensory quantities, the system is able to extract the cross-sensory relations and enforce, through mutual interaction, a plausible integration.

**Figure 8:**
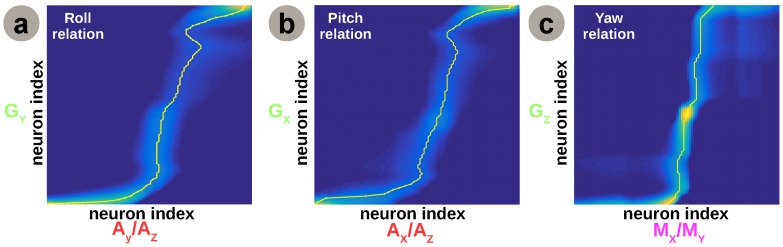
Network instantiation for 3D egomotion estimation: a decoupled view analysis. (**a**) Learned relation for roll; (**b**) Learned relation for pitch; (**c**) Learned relation for yaw. (yellow traces depict highest connection strengths).

In order to validate the extracted relations, we use the aforementioned mechanism to extract the roll, pitch, and yaw estimates for the quadrotor scenario. Figure 8 presents a decoupled view for each degree of freedom, depicting the learned relations.

**Figure 9:**
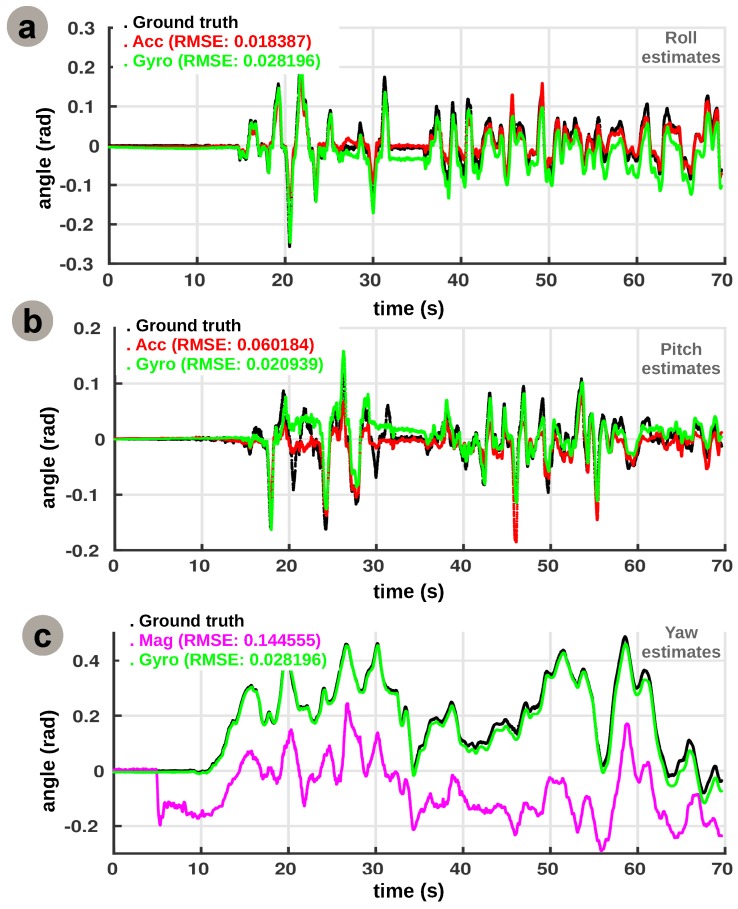
Network instantiation for 3D egomotion estimation: a decoupled view analysis. (**a**) Inferred sensory estimates for roll; (**b**) Inferred sensory estimates for pitch; (**c**) Inferred sensory estimates for yaw.

We observe that the learned relations resemble the nonlinear functions used in typical modelling approaches, although irregularities in the cross-sensory relations are preserved. More precisely, the agreement with the previous control approaches refers to the capability of the proposed system to extract the nonlinear transformations among sensory quantities, for example the transformation (i.e., trigonometric, arctangent) from a ratio among accelerations on *x* and *z* axis to an absolute pitch angle estimate. The learned cross-sensory relations, encoded in the Hebbian matrix, provide the intrinsic constraints between the sensors. After learning, in order to compute individual contributions of the sensors for each degree of freedom (e.g., roll), we feed one side of the network with samples from one sensory modality (e.g., accelerometer); we project the corresponding activity pattern through the learned Hebbian matrix to compute the cross-modal activation; and finally, we decode the expected value for the associated sensory modality (e.g., gyroscope). This process is performed for each of the associated sensory modalities in the network. The integrated and improved individual estimates are shown in Figure 9.

For roll estimation (Figure 9a) the network learns the relation between net rotational acceleration provided by the accelerometer and the absolute roll angle estimate provided by the gyroscope. Given that accelerometer data is noisy and gyroscope data drifts, as a consequence of the integration process, the network is able to “pull” the values of the two cues towards the correct value of the roll angle as given by ground truth (i.e., a sub-mm precision 3D motion caption system with infra-red cameras, Natural Point—OptiTrack). We evaluated the precision of the individual estimates using the Root Mean Squared Error (RMSE) as metric. Taking into account the metric, the accelerometer is within 2% of the correct value whereas the gyroscope estimates are within 3% of the ground truth.

For pitch estimation the network extracts the nonlinear dependency between the accelerometer data and the gyroscope data. Although both cues follow the trend of change in angle, as shown in Figure 9b, the accelerometer is overestimating, due to the noisy signal and the overall limited motion of the drone on this axis. The gyroscope contribution was able to modulate the accelerometer contribution such that the overall estimates are improved. Again, the accelerometer estimate is within 7% from the ground truth pitch angle whereas the gyroscope is more precise, with less than 3% deviation. Using only on-board sensory data the system can properly disambiguate its motion, as for example, due to occlusion the global tracking system might provide erroneous observations ($t_{1}$∼20 s and $t_{2}$∼30 s). In our setup, this will not have an impact on the estimates as we are using only on-board sensory data to estimate 3D egomotion and the external tracking data is just used as reference.

Finally, for yaw estimation the network uses the gyroscope absolute angle and the magnetometer contribution, based on magnetic field readings on the x and y axes. Interestingly, despite the fact that the yaw estimate of the magnetometer follows the trend, as visible in Figure 9c, there is an intrinsic offset of around 0.15 rad visible from *t* = 5 s. Investigating during many test flights, we noticed that the current change generated when starting the rotors introduced a significant modification in magnetic field distribution, subsequently reflected in the magnetometer readings. In the current setup, the inferred network is not able to explicitly compensate for the offset, as one can see in Figure 8c, where the Hebbian co-activation pattern is not as sharp as for roll and pitch. As a possible extension, the system could learn the offset in a separate map of the network and maintain a current estimate of relative offset of sensory modalities. These additional maps could furthermore refine the main cross-sensory relations through mutual interaction thus cancelling offset or bias effects, similar to our previous work in [15].

**Figure 10:**
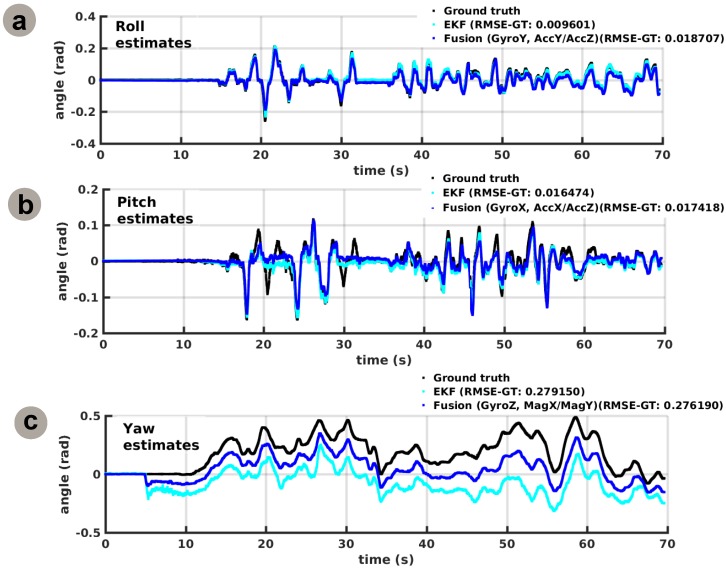
Network instantiation for 3D egomotion estimation: fused sensory data. (**a**) Fused estimate for roll; (**b**) Fused estimate for pitch; (**c**) Fused estimate for yaw.

Following the processing pipeline introduced by our approach, the system fuses the associated sensory cues for each degree of freedom using the learned relations and provides better estimates than the individual sensory cues, as shown in Figure 10. The fused estimates, based upon the learned relations, provide comparable performance with respect to ground truth with state-of-the-art approaches, typically used in quadrotor state estimation (i.e., EKF—Extended Kalman Filter [30]) as shown in Figure 10.

Our results show that the model is able to extract the underlying data statistics without any prior information such that the sensory data distribution was learned directly from the input data. Moreover, following the statistics of the data, the network allocates more neurons to represent areas in the sensory space with a higher density such that the cross-sensory relations are sharpened. As one can see there is no need for any specific parameter tuning routine to handle different kinds of input data for different scenarios.

The generic processing elements (SOM, HL) ensure that the network first learns the structure of the data in an unsupervised manner, and then uses this representation to sharpen its correlational structure. Moreover, given the learned relations, the network is able to infer missing quantities in case of sensor failures. As the relation is encoded as a synaptic weight, after learning, it is enough to provide samples from one sensor, encode them in the SOM, and project the activity pattern through the Hebbian matrix. The resulting activity pattern can be subsequently decoded to provide the missing real-world sensory value.

The proposed learning scheme extends our previous work, in which given various sensory inputs and simple relations defining inter-sensory dependencies, the model infers a precise estimate of the perceived motion. Now, by alleviating the need to explicitly encode sensory relations in the network, we propose a model providing flexible and robust multisensory fusion, without prior modelling assumptions, and using only the intrinsic sensory correlation pattern.

## 4. Discussion

The present work introduces a novel framework for learning underlying correlations in sensorimotor streams. As our preliminary results show, this approach has attractive features, highly relevant in today’s challenging and dynamic scenarios robotic systems operate in. Through self-creation, the system alleviates the need to manually perform tedious and constrained system identification and parametrization routines. Moreover, learning processes allow the system to extract the intimate (and not always regular) relations among correlated sensory cues.

After learning sensory relations from incoming sensory streams, the system supports judicious integration of the perceived quantities into a coherent percept that is more precise than individual sensory contributions, shown in Figure 9. In order to efficiently combine multiple contributions, the system extracts the distribution of the input sensory space such that more informative observations guide the integration process. The capability of extracting a sharp representation of inter-sensory correlations enables the system to learn the internal model as a quantification of the constraints the physics and the dynamics of the sensors impose on the system.

Furthermore, these constraints act upon acquired observations, such that individual estimates are “pulled” towards the correct and stable solution manifold, one which ensures consensus in the network and a precise fused estimate, as shown in Figure 10. Finally, the learned constraints provide a mechanism for fault-tolerance and prediction, allowing the system to internally construct a belief of the correct inter-sensory correlations based on the learned internal model and subsequent sensory observations, and discard outliers from unforeseen sensory events.

In order to emphasize the core contribution and significance of our model, we summarize the most important common features of other models (briefly introduced in the Introduction) following the same aspects we addressed when describing our model’s features.

One initial aspect is the design and functionality. Either using distributed representations, as in [4,5], or compact mathematical forms, as employed in [6,7], all methods encoded the input variables in a new representation to facilitate efficient computation.

A second aspect is the amount of prior information set by the designer in the system. It is typical that, depending on the instantiation, a new set of parameters is needed, making the models less flexible. Although less intuitive, the pure mathematical approaches like [6] need less tuning effort, due to the fact that the parameters are the result of an optimisation procedure. On the other side, the neurally inspired approaches, presented in [4,5,7] need a more judicious parameter tuning, as their dynamics are more sensitive, and can either reach instability or local minima. Prior information about inputs is generally needed together with knowledge about bounds of the sensory space and their probability distributions, tuning values, coefficients, or standardization processes.

A third aspect, relevant to the analysis, is the stability and robustness of the obtained representation. The inferred representation can be encoded in a weight matrix, as shown in [4,5], allowing a continuous refinement in the presence of new input samples, can be fixed in vector directions of random variables, as used in [6], or can be obtained as an optimisation process, as considered in [7].

The capability to handle noisy data, is another important aspect influencing applicability in real-world scenarios. Using either computational mechanisms for de-noising, as those employed in [4,5], the iterative updates to minimise a distance metric in [6], or optimisation techniques in [7], each method is capable to cope only with moderate amounts of noise.

Another relevant feature is the capability to infer (i.e., predict/anticipate) missing quantities once the relation is learned. The capability is available when using either the learned co-activation weight matrix, as done in [4,5], or the known standard deviations of the canonical variants in [7] and is not available in [6].

Although not explicitly treated in the presented models, decoding the extracted representations is not trivial. Using a tiled mapping of the input values along the neural representations the model proposed in [4] decoded the encoded value in activity patterns by simply computing the distribution of the input space over the neural population units, while work in [5] used a simple winner-take-all readout, given that the representation was constrained to have a uniquely defined mapping. The model in [7] learned the relations in data space through optimisation processes. This allowed to simply project available sensory values through the learned function to infer the second variable preserving scale. Albeit its capability to precisely extract nonlinear relations from high-dimensional random datasets, the model in [6] couldn’t provide any readout mechanism to support a proper decoded representation of the extracted relations. This is due to the fact that the method cannot recover the sign and scale of the relations.

## 5. Conclusions

Given the complex and multimodal scenarios in which robotic systems operate, with noisy and partially obstructed environment features, the capability to precisely and rapidly extract estimates of egomotion critically influences the set of possible actions. With extensive sensing capabilities, today’s robotic systems face a different problem, namely how to advantageously use correlated sensory streams for building an internal model of their motion and environment, which can subsequently improve their perception. Although inherently noisy and uncertain, available sensory streams contain regularities imposed by the motion of the robot through the environment.

In the challenge to understand how a robotic system can exploit these underlying regularities and correlations, we developed a framework capable of tackling three key topics related to the fundamental understanding of sensorimotor learning: what has to be learned, how is it learned, and how knowledge developed during learning can be represented and used in closing the loop. In order to respond to the first topic, namely what has to be learned, we developed a mechanism which uses generic information metrics, like entropy, to analyse the intimate structure of the incoming sensory streams, identify regularities, and use them to build a network of sensory variables corresponding to each of the available sensors. After a structure is created, the system uses simple, well understood, and cheap computations to learn the intrinsic relations among sensors along with their statistics. Finally, in order to use the knowledge about sensory correlations acquired during learning, the system implements the extracted relations as constraints to fuse the available sensory streams so as to obtain a globally more precise state estimate.

Going away from traditional approaches, our perspective resides on four core principles also known to explain development processes in biological systems. Given the wide range of available sensory streams, the system learns sensory associations from the data and builds a network of associations. This network enforces relevant rules for multisensory fusion. Exploiting underlying regularities in the data, the system uses a distributed representation and simple computations, like competition and cooperation to learn the underlying correlations. Finally, the development process of the system is autonomous and it is not mediated by a teacher or error/reinforcement signal.

Amenable to support analysis and versatile modelling, these principles enable the system to capture the full complexity of real-world motion estimation and we expect that the learning model will generalize to new tasks.

This initial exploratory phase allowed us to investigate the flexibility and robustness of neurally inspired computation mechanisms for representation, learning, and processing of sensorimotor streams. In the next steps, we aim at closing the loop after learning the sensory feedback, and further explore re-learning and adaptation for new scenarios. Moreover, focusing on having a real-time capable system after deployment (i.e., after learning sensory associations and correlations), we are interested in investigating parallel implementations. This is a natural extension to exploit inherent distributed representations and computation in our system, especially on parallel embedded hardware platforms. Finally, due to knowledge about network structure and the simple dynamics, the system can be easily extended to accommodate an arbitrary number of sensory modalities and diverse representations for improved perception.
